# Enhancing touch sensibility with sensory electrical stimulation and sensory retraining

**DOI:** 10.1186/s12984-024-01371-4

**Published:** 2024-05-16

**Authors:** Eduardo Villar Ortega, Karin A. Buetler, Efe Anil Aksöz, Laura Marchal-Crespo

**Affiliations:** 1 Motor Learning and Neurorehabilitation Laboratory, ARTORG Center for Biomedical Engineering Research, University of Bern, Bern, Switzerland; 2 rehaLab-The Laboratory for Rehabilitation Engineering, Institute for Human Centred Engineering HuCE, Division of Mechatronics and Systems Engineering, Department of Engineering and Information Technology, Bern University of Applied Sciences, Biel, Switzerland; 3 Department of Cognitive Robotics, Delft University of Technology, Delft, The Netherlands; 4 Department of Rehabilitation Medicine, Erasmus University Medical Center, Rotterdam, The Netherlands

**Keywords:** Electrostimulation, Sensory training, Robotic neurorehabilitation, Virtual reality, Electroencephalography, Alpha Power

## Abstract

A large proportion of stroke survivors suffer from sensory loss, negatively impacting their independence, quality of life, and neurorehabilitation prognosis. Despite the high prevalence of somatosensory impairments, our understanding of somatosensory interventions such as sensory electrical stimulation (SES) in neurorehabilitation is limited. We aimed to study the effectiveness of SES combined with a sensory discrimination task in a well-controlled virtual environment in healthy participants, setting a foundation for its potential application in stroke rehabilitation. We employed electroencephalography (EEG) to gain a better understanding of the underlying neural mechanisms and dynamics associated with sensory training and SES. We conducted a single-session experiment with 26 healthy participants who explored a set of three visually identical virtual textures—haptically rendered by a robotic device and that differed in their spatial period—while physically guided by the robot to identify the odd texture. The experiment consisted of three phases: pre-intervention, intervention, and post-intervention. Half the participants received subthreshold whole-hand SES during the intervention, while the other half received sham stimulation. We evaluated changes in task performance—assessed by the probability of correct responses—before and after intervention and between groups. We also evaluated differences in the exploration behavior, e.g., scanning speed. EEG was employed to examine the effects of the intervention on brain activity, particularly in the alpha frequency band (8–13 Hz) associated with sensory processing. We found that participants in the SES group improved their task performance after intervention and their scanning speed during and after intervention, while the sham group did not improve their task performance. However, the differences in task performance improvements between groups only approached significance. Furthermore, we found that alpha power was sensitive to the effects of SES; participants in the stimulation group exhibited enhanced brain signals associated with improved touch sensitivity likely due to the effects of SES on the central nervous system, while the increase in alpha power for the sham group was less pronounced. Our findings suggest that SES enhances texture discrimination after training and has a positive effect on sensory-related brain areas. Further research involving brain-injured patients is needed to confirm the potential benefit of our solution in neurorehabilitation.

## Introduction

Cerebral stroke, a major cause of persisting and long-term disability [1], often leads to somatosensory impairments [2] with overall prevalence rates between 34–84% [3]. Somatosensory impairment hinders the interpretation of somatosensory information—e.g., identification of movements, detection of touch, discriminating between stimuli—, hampering individuals’ quality of motor control, and therefore, their safety and performance in activities of daily living and independence.

Despite the high prevalence and negative impact of somatosensory impairments on patients’ lives, somatosensory interventions are typically overlooked in neurorehabilitation compared to motor training [2, 4–6]. Somatosensory interventions are therapeutic techniques that clinical practitioners employ to retrain loss of body sensation. Somatosensory interventions have been classified into three types: sensory retraining, sensory stimulation, and hybrid interventions [7]. *Sensory retraining* techniques such as two-point discrimination and the tactile discrimination test (TDT) are commonly used in clinical settings. However, the effects of sensory retraining in neurorehabilitation are still unclear and there is only scarce evidence to support their use to improve hand function [4, 8]. The application of *sensory stimulation*—i.e., repetitive exposure to stimuli without verbal or attentional focus [9]—such as electrical, thermal, and vibrotactile stimulation has been shown to be effective in enhancing and recovering somatosensory and motor functions [2, 7, 10, 11]. For example, sensory electrical stimulation (SES) is widely accepted to induce rapid plastic changes in the excitability of the motor cortex [12] and changes in synaptic transmission and plasticity in the somatosensory cortex [13]. Consequently, there has been a growing interest in using SES in clinical settings [14], as it has been shown to moderately improve hand function, dexterity, and motor training after stroke [4, 7, 14], improve sensorimotor function [15], and modulate cortical oscillations [16]. Yet, the sole application of electrical stimulation as a passive treatment does not seem to be as effective as when applied simultaneously with active movements, i.e., *hybrid intervention* [7, 11, 14, 17–24].

Typically, SES is provided at intensities below or above the sensory threshold—i.e., the stimulus intensity in which the user feels the stimulus without evoking any motor responses [11]. Sensory electrical stimulation is also provided using different types of stimulation protocols, such as transcutaneous electrical nerve stimulation (TENS), whole-hand electrical stimulation (WH stimulation)—also called glove stimulation—, repetitive sensory stimulation (RSS), or peripheral nerve stimulation (PNS). The stimulation protocols often differ in electrode location, types of electrodes, and electrostimulation parameters employed. The efficacy of SES in enhancing somatosensory function and motor function is related to the optimal combination of stimulation parameters, such as stimulation intensity, frequency, stimulus pattern, pulse duration, and electrode location. These parameters are currently being investigated concerning sensorimotor behavior, neurophysiological markers during tactile interventions [25], and their role in inducing cortical plasticity [12].

Especially promising is whole-hand electrical stimulation as it has been shown effective in depolarizing afferent fibers across the hand, enhancing the excitability of both sensory and motor mechanisms without relying on specific electrode placements, thus making it practical, side-effect free, and readily compatible with other interventions [26]. It has been shown that 50 Hz and 2 Hz whole-hand electrical stimulation frequencies, combined with a pulse width of 0.30 ms, are particularly effective in inducing lasting neuromodulatory changes and corticomotorneuronal effects in the primary motor cortex [13]. These effects were found to be reinforced at intensities above the motor threshold (with 2 Hz) and sensory threshold (with 50 Hz) [27]. A study using functional magnetic resonance imaging (fMRI) found that 30 min of whole-hand stimulation increased the oxygen level-dependent (BOLD) responses and modulated the corticospinal excitability and intracortical inhibitory and excitatory circuits, investigated using transcranial magnetic stimulation (TMS) [13]. During whole-hand SES, the depolarization of group Ia (primary large muscle afferents) and Ib (joint receptors), and group II (slow and fast adapting skin receptors) afferents in the hand led to synchronous tonic input to the brain [13]. Moreover, a study by Peurala et al. demonstrated that sensory subthreshold stimulation primes cortical networks and increases brain activity during motor tasks [15].

While it has been shown that both sensory retraining and electrical stimulation techniques could facilitate adaptive neuroplastic changes [28, 29], there is a series of limitations associated with each technique. First, sensory retraining often requires patients to physically move their limbs, limiting such interventions to patients with mild motor impairment. For example, during the TDT—a conventional sensory retraining intervention to treat loss of touch sensitivity—, patients are asked to tactually explore a set of grating textures while a physiotherapist assists them in moving their limbs as needed. Thus, such intervention requires the therapists to continuously support the patient, which can be time-consuming, labor-intensive, and potentially less effective for patients with severe motor impairments [30]. Second, the effect of sensory stimulation on stroke recovery in the early stages of the rehabilitation process is unclear because most studies investigating the effects of sensory stimulation only involved patients in the chronic stage [14]. This may be due to a lack of instruments that can provide sensory stimulation during sensory retraining interventions while simultaneously assisting patients with motor impairment in the acute phase.

To address these limitations and study the effect of sensory electrical stimulation on sensory retraining, we present a novel *hybrid intervention* that combines robotic assistance to physically assist individuals during sensory retraining—i.e., discriminating textures haptically rendered by the robotic device—with whole-hand sensory electrical stimulation. During sensorimotor training, robotic devices can deliver precise, reproducible stimuli and physical guidance as needed. The potential use of robotic devices to provide sensory retraining interventions for proprioception and tactile discrimination in healthy individuals and stroke survivors has been already demonstrated [31–37]. Additionally, the use of robots has been shown to be useful when combined with vibrotactile feedback as conscious haptic cues to enhance proprioceptive acuity and motor performance [38], and reduce upper limb spasticity [39]. While some studies have combined electrical neurostimulation with robotic training, most of these employed neuromuscular or functional electrical stimulation (FES)—i.e., electrical stimulation techniques that directly stimulate nerves and muscles to generate movements—which goes beyond the sensation threshold intensity. However, stimulation intensities below the motor threshold target sensory afferents, overcoming the limitations of FES, which include among others the recruitment of motor axons in a non-physiological manner that may cause pain and discomfort [40, 41] and may lead to an afferent blocking effect, i.e., affecting the transmission of somatosensory information back to the central nervous system [42]. Moreover, SES has been found to provide additional functional improvements in chronic stroke patients who have reached a plateau in the recovery process, particularly when it is in tandem with voluntary efforts [43]. Only a few studies have used SES as an unconscious sensory input to the central nervous system (CNS) combined with upper limb robotic rehabilitation. For example, Capone et al. combined transcutaneous vagus nerve stimulation with robotic rehabilitation and found that the treatment group improved upper limb function after stroke compared to the sham stimulation group [44]. However, the use of SES in the peripheral nerves (e.g., whole-hand stimulation), including both at and below the sensory threshold, in combination with robotic rehabilitation is still in its early stages.

Importantly, the effects of somatosensory interventions on brain activity have yet to be extensively explored. Gaining a better understanding of the underlying neural mechanism and dynamics associated with sensory retraining and sensory stimulation could ultimately enhance the efficacy of neurorehabilitation interventions. To identify neurophysiological markers that characterize sensory retraining during SES, we employed electroencephalography (EEG). EEG is a non-invasive and cost-effective neuroimaging technique that measures electrical activity of the brain by means of electrodes mounted on the scalp. Due to its excellent temporal resolution, EEG has been widely used to study brain functions. Literature in EEG suggests that the alpha frequency band (8–13 Hz) activity is associated with sensory processing [45] and several studies have examined the dynamics of alpha oscillations following SES. For example, in a study by Yıldırım et al., the authors investigated the modulatory effects of TENS on alpha power as neural markers of sensory decline in healthy young and elderly participants [45]. They found that healthy young participants, compared to their elderly counterparts, increased alpha activity in response to the stimulation. Additionally, Tu-Chan and colleagues [16] investigated the usage of SES to facilitate the recovery of hand function and found that it was associated with improvements in hand dexterity, as well as changes in cortical oscillations. In their study, changes in the ipsilesional motor theta and alpha power were significantly correlated with finger individuation improvements [16]. Furthermore, in the field of neural prosthetics, several researchers have studied neural markers during sensory retraining interventions, particularly in tactile discrimination tasks [46–49]. For instance, Su et al. [48] found that alpha band attenuation in the somatosensory cortex was correlated with tactile discrimination performance.

This study aimed to examine the effectiveness of whole-hand sensory electrical stimulation in enhancing tactile discrimination training and its associated effects on brain activity in healthy participants, as a first step toward future applications in stroke rehabilitation. We ran a single-session parallel design experiment with 26 healthy participants who passively explored—i.e., physically guided by a robotic device—and discriminated the odd texture from a set of three visually identical virtual textures rendered using a robotic device [50, 51]. The study included three experimental phases: pre-intervention, intervention, and post-intervention. During the intervention phase, the treatment group received whole-hand SES subthreshold stimulation, i.e., below the threshold of sensory perception, while the control group received sham stimulation. In addition, EEG was employed to measure the changes in brain activity within experimental phases and between the groups, relative to the pre-intervention. Specifically, we assessed changes in alpha power—i.e., time–frequency representation (TFR) of power—, strength—i.e., global field power (GFP), a measure of the overall electrical activity at the scalp—, and brain networks configurations—i.e., global map dissimilarity (GMD), an index of topographic differences in brain activity patterns.

We hypothesized that participants who received whole-hand SES during training would demonstrate significantly better texture discrimination during and after the intervention, relative to pre-intervention, compared to those who did not receive SES. Similarly, we expected that participants training with SES would show stronger changes in sensory brain activity (i.e., alpha power) and learning-related neural adaptation (GMD) relative to pre-intervention compared to the sham group. Furthermore, we expected that both groups would show improvements in texture discrimination during and after the intervention, relative to pre-intervention. Similarly, we expected that both groups would show modulations in sensory brain activity during and after the tactile discrimination training versus before the training.

## Methods

### Participants

Twenty-six healthy participants (11 females) with ages ranging from 23 to 43 (mean of 30.15 years, ± 5.28 standard deviation), were recruited to participate in the study. All participants gave written consent to participate in the study, which the local ethical committee (Swiss Cantonal Ethics Committee; Basec ref: 2018-01179) and the Swiss Agency for Therapeutic Products (Swissmedic ref: 100000432) approved, and that complied with the Declaration of Helsinki. All participants were right-handed according to the Edinburgh Handedness Inventory [52].

The sample size was determined using the *R* package *sensR* [53]. We employed results from a previous experiment performed with a similar setup in our calculations [51], a desired power of 0.80, a type I error equal to 0.05, and a probability of guess of 1/3, specifically, using the triangle test. This resulted in a sample size estimation of a minimum of 12 participants per experimental group.

### Experimental set-up

#### Robotic setup

**Fig. 1:**
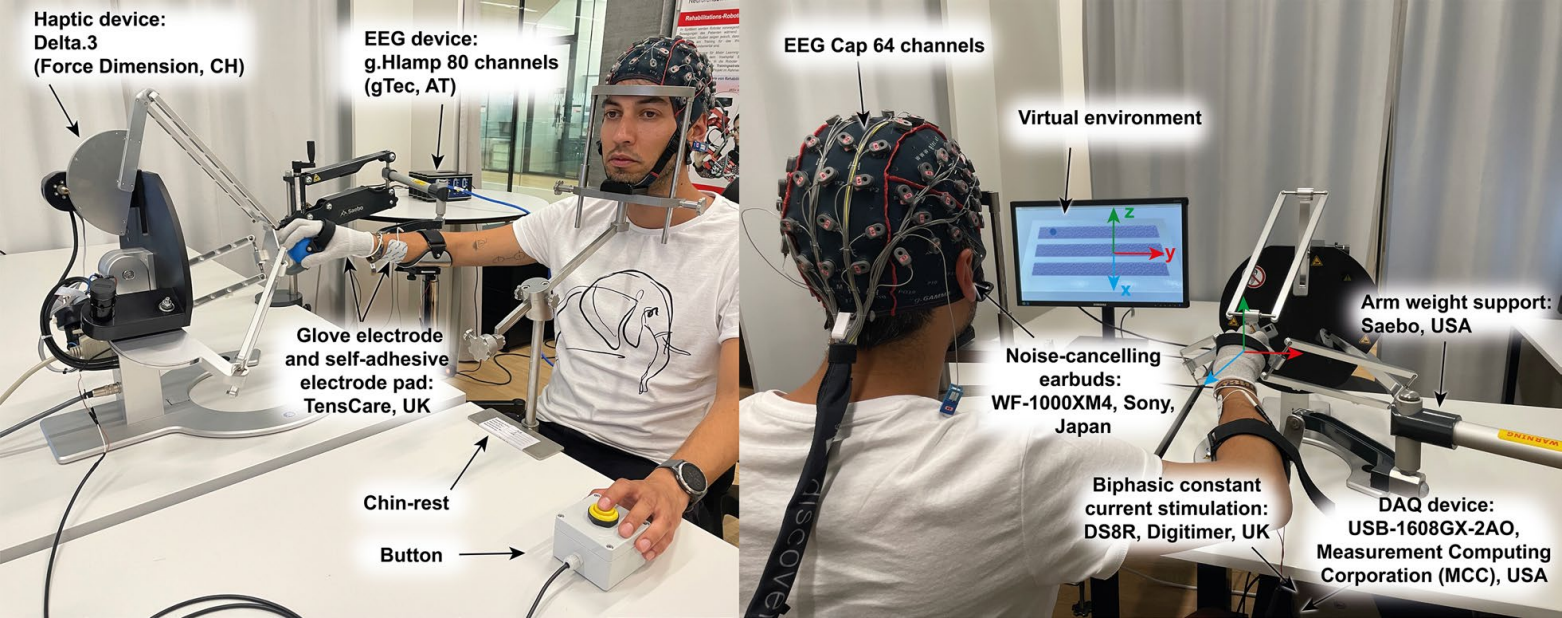
Experimental set-up. The Delta.3 robot was placed on a table to the right of the participant. An arm weight support mechanism was attached to the table and was employed to support the participant’s arm weight during the experiment to reduce fatigue. Participants placed their chin on a chin rest attached to the table in front of them. A LED monitor placed on the table in front of the participant showed the virtual environment. After the first familiarization phase, a curtain was attached from the chin rest to the monitor (not shown here) to hide the robot and the participant’s hand from the participant’s sight. Participants wore noise-canceling headphones to mask potential noises from the robot actuators

The experimental set-up (Fig. 1) consisted of a 24 inch monitor (S24E650, Samsung, South Korea), a robotic device (Delta.3, Force Dimension, Switzerland), noise-canceling headphones (WF-1000XM4, Sony, Japan), and a custom-made response box with a push-button. The participant’s hand was attached to the robot end-effector by using a Velcro® strap. A passive arm weight support system (SaeboMAS mini, Saebo, USA) was used to reduce arm fatigue during the experiment. The passive arm weight support device was secured to the participants’ forearms using a Velcro® strap and the amount of weight supported was adjusted for each participant at the beginning of the experiment and kept constant during the experiment. Participants wore noise-canceling headphones, which masked the potential noises from the robot actuators.

#### Sensory electrical stimulation

SES was provided using a constant current stimulator device (DS8R, Digitimer, UK). A data acquisition device (DAQ) (USB-1608GX-2AO, Measurement Computing Corporation, USA) was used to trigger the constant current stimulator via the transistor-transistor logic (TTL) synchronization input. Participants wore two electrodes on their dominant hand: a mesh-glove (iglove, TensCare, UK)—anode—and a self-adhesive electrode (TensCare, UK, 50 × 90 mm)–cathode. To accommodate for different hand sizes, electrodes were available in two sizes, medium and large. The self-adhesive electrode was placed on top of the median and ulnar nerves over the dorsal surface of the forearm proximal to the wrist joint and separated at least 2 cm from the glove and the cuff of the weight support device. This method aligns with the approach suggested by Dimitrijevic, which indicates that the effectiveness of mesh-glove stimulation is not critically dependent on the exact location of the surface electrodes [26].

#### EEG system

EEG was recorded using an 80 bio-signal amplifier (g.HIAMP, g.tec medical engineering GmbH, AT). The participants wore a 64 channels EEG cap (g.GAMMAcap2, g.tec medical engineering GmbH, AT) with active electrodes (g.SCARABEO, g.tec medical engineering GmbH, AT) that was mounted centrally on the head using the distance between the nasion and inion and the distance between the right and left preauricular points of the participants. The location of the *CZ* electrode was below the intersection of both distances. EEG trigger events were registered by synchronizing the DAQ digital outputs and the g.HIAMP digital inputs via a custom-made cable. Seven digital outputs from the DAQ were employed to register four experiment phases: familiarization, pre-intervention, intervention, and post-intervention; and two events: the start and end of a trial.

### Experimental task

The task consisted of discriminating the odd virtual texture from three visually identical textures—haptically rendered using the haptic device—using indirect touch by holding the robot end effector. Participants were asked to passively explore (i.e., physically guided by the robot) the virtual textures and select the odd texture by pressing a custom-made button using their non-dominant hand while they were on top of the texture they felt was different from the other two.

#### Virtual textures

The haptic implementation of the virtual textures (tactile stimuli) is described in detail in [51]. We only provide here a summary for completeness.

**Fig. 2:**
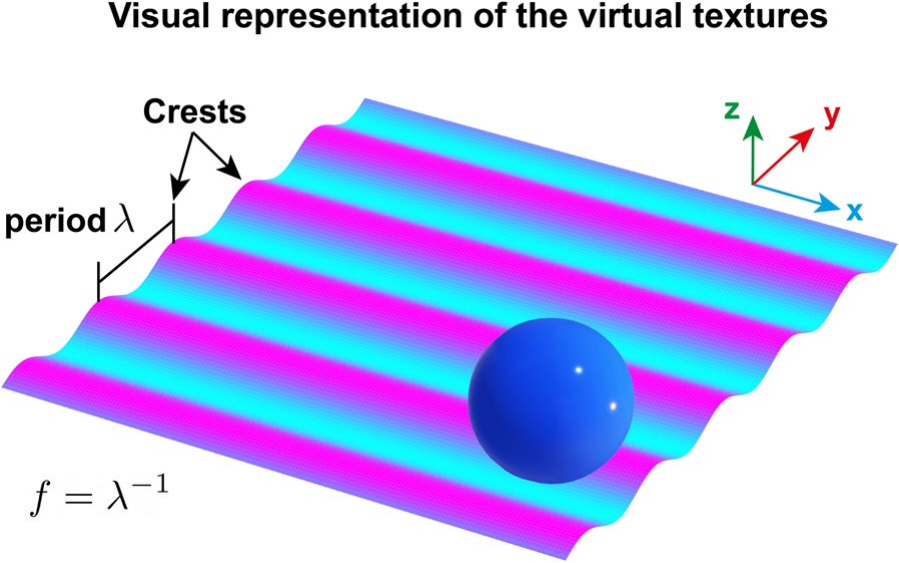
Tactile stimuli: the haptically rendered textures. The virtual textures were generated following sinusoidal gratings of spatial frequency *f*. The spatial frequency is defined as the inverse of the distance of two adjacent crests $\lambda$. Please note that the size and proportions of the ball (representing the end-effector position of the robot) and texture are for illustration purposes, and they do not match the sizes used in the experiment. For more information, please refer to [51]

The virtual textures were composed of sinusoidal gratings rendered along the robot end-effector *y*-axis (lateral direction), as represented in Fig. 2. Participants haptically felt these gratings when in contact with the texture and while they moved the robot end-effector along the *y*-axis as forces of magnitude:

1 $$\begin{aligned} F_g=C\sin {(2{\pi }{f}{y_{EE}})}. \end{aligned}$$

Thus, the magnitude of the interaction forces $F_g$ depended on the constant *C* = 3 N, the robot end-effector position along the *y*-axis (${y_{EE}}$) and the spatial frequency *f* of the gratings (see Table 1). The spatial frequency is defined as the inverse of the distance between two sequential crests (Fig. 2). No forces were applied along the *x*-axis.

**Table 1:** Stimulus set for the rendered textures

${f_{St}}$ ($m^{-1}$)	${f_{Co}}$ ($m^{-1}$)			
	More coarse		Less coarse	
164	120	142	186	208

We kept constant the spatial frequency of the standard stimulus (${f_{St}}$) while in each trial the spatial frequency of the comparison stimulus (${f_{Co}}$) varied along more coarse and less coarse textures

The virtual textures were rendered on top of a haptic table of 0.20 m × 0.10 m. This virtual table was also haptically rendered following the equation [51]:

2 $$\begin{aligned} F_{z} = {\left\{ \begin{array}{ll} K_{z} (z_{tbl}-z_{EE}) + B_{z}(\dot{-z_{EE}}) &{} \text {if } {z_{EE}} {<} {z_{tbl}} \\ 0 &{} \text {otherwise}, \end{array}\right. } \end{aligned}$$

where $F_z$ is the magnitude of the force along the *z*-axis (vertical) that depends on the relative position and velocity of the robot end-effector in the *z*-axis ($z_{EE}$, $\dot{z_{EE}}$) w.r.t. the height of the virtual table $z_{tbl} = 0.001$ m, with gains ${K_{z} = 1960}$ N/m and ${B_{z} = 28}$ Ns/m.

Participants explored five different virtual textures during the experiment that differed in their spatial frequency, as shown in Table 1. A standard stimulus $f_{St}$ and four different comparison stimuli $f_{Co}$ were included in the tactile discrimination task. The standard stimulus (164 $m^{-1}$) remained constant throughout the experiment, whereas the comparison stimuli (120, 142, 186, and 208 $m^{-1}$) varied between trials. To ensure comprehensive randomization and prevent potential learning effects, both the $f_{Co}$ and the position of the standard stimulus $f_{St}$ within the triplet were randomized in each trial. The order of stimuli presentation followed the method of constant stimuli. The sensory discrimination test employed was the method of triangles. During each trial, the textures were presented in triplets with combinations of St/Co/Co, Co/St/Co, Co/Co/St, St/St/Co, St/Co/St, and Co/St/St, ensuring a balanced and unbiased assessment of tactile discrimination.

#### Robotic assistance

Participants were physically assisted during the exploration of the virtual textures via haptic guidance provided by the Delta robot. The magnitude of the haptic guidance force $F_{hg}$ was computed using a Proportional Derivative (PD) controller described as:

3 $$\begin{aligned} F_{hg} = {\left\{ \begin{array}{ll} \ddot{y}_R + B_{hg}({\dot{y}}_R-{\dot{y}}_{EE}) + K_{hg}(y_R-y_{EE}) &{} \text {if } {contact} \text { is } {True} \\ 0 &{} \text {otherwise}, \end{array}\right. } \end{aligned}$$

where ${y_{EE}}$ and ${{\dot{y}}_{EE}}$ are respectively the robot end-effector position and velocity in the lateral *y*-axis, i.e., along the perceived textures. The stiffness and damping coefficients were set to ${K_{hg}}$ = 300 N/m and ${B_{hg}}$ = 60 Ns/m, respectively. The cycloidal motion law was used to compute the enforced desired trajectory—${\ddot{y}_R}$, ${{\dot{y}}_R}$, and ${y_R}$—, as described in [51].

Participants were instructed to allow the robot to guide them and not resist the haptic assistance. Transitioning between textures while the assistance was on was possible by leaving the texture through one of its sides or by lifting the end-effector and breaking contact with the virtual table.

The total forces applied by the robotic device during the texture exploration task are thus:

4 $$\begin{aligned} \vec {F}_{Total}=F_{rd}\vec {j}+F_{z}\vec {k}+F_{hg}\vec {j}, \end{aligned}$$

where $\vec {j}$ and $\vec {k}$ are unit vectors along the *y*- and *z*-axis, respectively.

### Experimental procedure

The experiment took place in a quiet and isolated area in the Motor Learning and Neurorehabilitation Laboratory at the Swiss Institute for Translational and Entrepreneurial Medicine (SITEM-Insel), Bern, Switzerland, under the supervision of the experimenter, i.e., the engineer who developed the system and first author of this article. During the experiment, the individual participants sat on a comfortable chair with a backrest. A head-chin rest was adjusted according to each participant’s height and employed to prevent head movements and thus motion artifacts in the EEG. Once the chin rest was adjusted, we placed the EEG cap and the whole-hand stimulation electrodes. We then adjusted the weight support according to each participant’s arm weight and attached the participant’s hand to the robot end effector. Then, we fixed the participant’s position by locking the chair’s wheels. Using a Velcro® strap, we secured the electro-connector box (g.tec) to the back of the chair. Finally, a curtain was placed from the chin-rest to the monitor using Velcro® dots, thus hiding the robot and the participant’s hand from her/his sight. We applied Signaspray Electrode Spray (Parker Laboratories, USA) to the participant’s dominant hand after the setting up of the EEG and just right before starting the experiment to improve the skin-electrode conductivity.

**Fig. 3:**
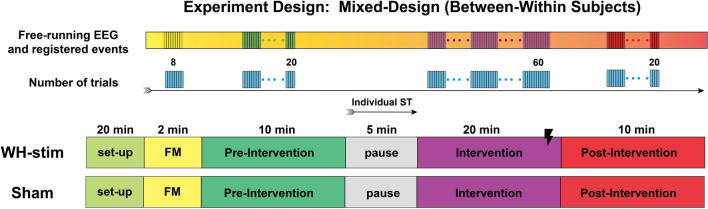
Experimental design. Participants completed a single-session experiment. The session included familiarization (FM), pre-intervention, intervention, and post-intervention. We used the pause before the intervention to identify the participant’s individual sensory threshold (ST). Half of the participants were randomly allocated to the WH-stim group, receiving somatosensory stimulation during the intervention, whereas the second half received sham stimulation (stimulation with 0 mA)

The experimental design is illustrated in Fig. 3. We randomly allocated half of the participants to the whole-hand stimulation (WH-Stim) and the other half to the Sham group. The group randomization was performed using Research Randomizer (Version 4.0) [54]. Participants were not informed about their group allocation. Participants performed the experiment in a single session, which was subdivided into four phases: familiarization, pre-intervention, intervention, and post-intervention.

#### Familiarization (FM)

The experiment started by visually presenting a virtual texture to the participants. We presented a single virtual texture (*f*=100 m^-1^), which included visual information about the spatial frequency, i.e., showing in dark color the grooves and in light color the crests of the texture gratings, to facilitate the understanding of the haptic stimuli. Participants were free to explore this first texture. We then presented eight trials in which the texture combination was always of the form A/B/B, with spatial frequencies ${f_A}$ = 100 m^-1^, and ${f_B}$ = 164 m^-1^. During these eight familiarization trials, we asked participants to select the odd texture among the three visually identical textures by pressing the custom button with their left hand when on top of the texture they considered different. We did not randomize the location of the odd texture across these familiarization trials, as the only purpose was to let the participants get familiar with the robot and learn how to move around the textures. Participants performed the familiarization with the robotic assistance on and without the curtain in place. The familiarization lasted approximately 2 min.

#### Pre- and post-interventions

During the pre- and post-interventions, participants did not receive SES. Participants performed 20 consecutive trials while physically guided by the robot during the texture exploration. The order of the presented stimuli from Table 1 was randomized once during the pre-intervention and the same order was repeated during the intervention and post-intervention. During the block of 20 trials, each comparison stimulus *Co* was presented five times. Each pre- and post-intervention lasted approximately 7–10 min.

#### Determining the participants’ individual sensory threshold

Shortly after the pre-intervention, participants were allowed to rest for 5 min. During this pause, the individual sensory threshold intensity was determined for all participants, independently of the group they were randomly allocated to.

**Fig. 4:**
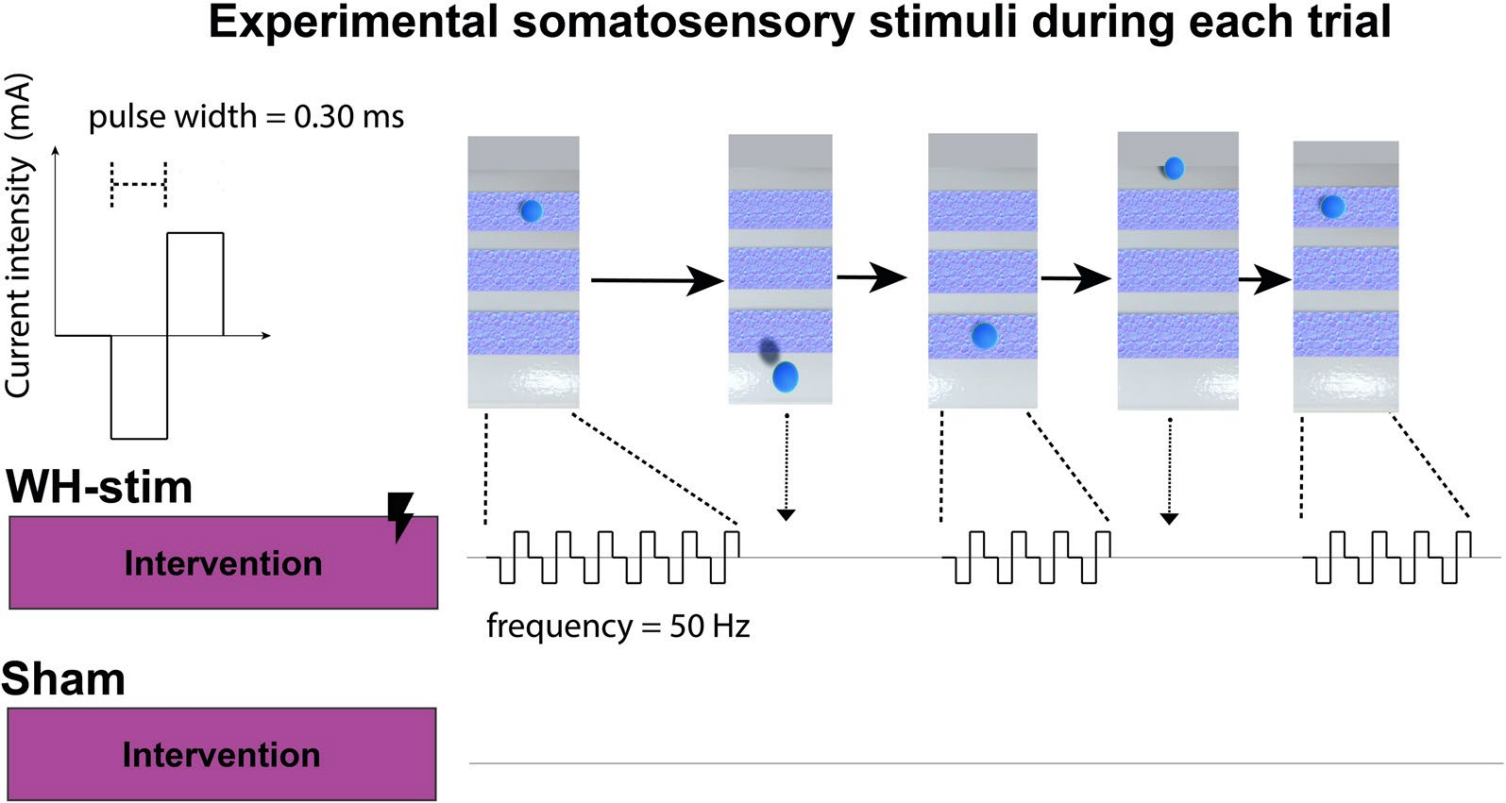
Somatosensory electrical stimulation. Participants from the whole-hand stimulation group (*WH-Stim*) received somatosensory electrical stimulation during the intervention, whereas the *Sham* group received 0 mA stimulation. The stimulation was provided only when participants were within any of the textures. The stimulation train frequency was set to 50 Hz. Please note that the 10 μs interphase delays are not visually represented in this figure

The somatosensory stimulation signals were rectangular charge-balanced biphasic with 10 μs delays, cathode-first with 300 μs pulse duration and provided in a fix 50 Hz rate as shown in Fig. 4. Charge-balanced biphasic waveforms were selected as these protocols are commonly used in neural stimulation studies to prevent tissue damage due to charge accumulations [55]. The individual sensory threshold intensity was determined by manually increasing the intensity of the electrical stimulation, starting from 0 mA, in increments of 0.5 mA until a tingling sensation due to the stimulation was perceived by the participant. We chose increments of 0.5 mA based on existing literature, which suggests that the sensory threshold for most participants lies between 2 and 4 mA [13], to reach the expected threshold quicker.

The stimulation intensity was then reduced using decrements of 0.2 mA until the stimulation was no longer perceived. We then increased the intensity in increments of 0.1 mA, i.e., the smallest increment allowed by the Digitimer, until the stimulation was perceived again. This final intensity value was considered the sensory threshold, which varied between participants (mean 2.80 mA ± 0.74 mA standard deviation).

#### Intervention

During intervention, participants received whole-hand sensory electrical stimulation (WH-Stim) or sham stimulation (i.e., no stimulation) during the texture discrimination task. The stimulation intensity was set to 95% of the individual sensory threshold for the *WH-Stim* and to 0 mA for the *Sham* group. To prevent participants from becoming overly focused on the sensation of electrical stimulation, which might detract from their primary task of discriminating virtual textures, we avoided using the sensory threshold intensities or higher. The rest of the stimulation parameters were kept the same as the ones used during the individual sensory threshold determination. The electrical stimulation was delivered only when participants in the intervention group were in contact and inside one of the three textures, while 0 mA was provided otherwise.

The intervention block included 60 trials grouped into three blocks of 20 trials each. Each 20-trial block followed the stimuli order determined during pre-intervention. In total, the intervention blocks lasted approximately 20–25 min. Participants were informed that they might or not perceive the electrical stimulation during the intervention.

### Data processing

#### Behavioral data

The *task performance* was evaluated using the *probability of correct responses*. The correctness of the response ${Y_i}$ to the discrimination of the odd texture was registered on each trial *i* following the formula:

5 $$\begin{aligned} Y_i = {\left\{ \begin{array}{ll} 1 &{} \text {if response was correct} \\ 0 &{} \text {if response was incorrect}. \end{array}\right. } \end{aligned}$$

We then used ${Y_i}$ to compute the probability of correct responses by dividing the number of correct responses after 20 trials by the number of trials as shown in Eq. 6.

6 $$\begin{aligned} p = \frac{1}{20}\sum \limits _{i=1}^{20} Y_i \end{aligned}$$

We also evaluated several *kinematic measures* related to the participants’ texture exploration behavior. In particular, we recorded the *scanning time*, representing the average exploration time per trial across all the textures while participants were in contact with the textures and moving faster than 0.01 m/s. We also calculated the *path length* along the *y*-axis, expressed in meters, as the distance covered during the exploration of textures, averaging the three textures for each trial. Finally, we calculated the *scanning speed*, denoted in meters per second along the *y*-axis, indicating the average speed during texture exploration.

#### Electrophysiological data

The electrophysiological data were segmented into epochs based on two trigger events: the start and end of each trial. The start epochs ranged from the moment the trial began (0.0 s) to 1.4 s post-trigger onset, while the end epochs ranged from 1 s before the end of the trial (i.e., when participants pressed the response button) to 0.4 s after it. We used these two trigger events because they allowed us to investigate potentially different brain processes associated with preparation and decision-making in sensory processing.

To characterize the effects of SES on sensory retraining, we performed global electric field analyses in the alpha frequency domain (i.e., in the range of 8–12 Hz), the global field power (GFP), and the global map dissimilarity (GMD). Global electric field analysis examines the electric field at the scalp and aims at differentiating between the effects caused by quantitative changes in response strength of statistically indistinguishable brain generators and qualitative changes in the topographic configuration of these generators [56–58]. This approach is also known as electrical neuroimaging and provides several advantages over traditional single-electrode waveform analyses because it is reference-independent and avoids experimenter biases, i.e., this approach does not require a priori hypotheses about electrode location(s) or period of interest (POI) at which effects might be expected [59, 60].

#### Preprocessing

The EEG data were sampled at a rate of 1200 Hz and preprocessed offline using the Python packages *MNE* [61] and *Autoreject* [62], and following an established preprocessing pipeline [63]. Electrode impedances were kept below 100 k$\Omega$. Two channels (i.e., ${F_{z}}$, and ${O_{z}}$ according to the extended 10–20 EEG coordinate system) were identified as bad channels in all subjects due to technical deficiencies, and therefore, excluded from preprocessing and analyses.

The preprocessing consisted of removing power-line interference (i.e., 50 Hz and its harmonics), recalculating the signal against the average reference and band-pass filtering (with 0.1 Hz cutoff frequency and low-pass of 40 Hz) using the function *filter* provided by *MNE*. Further, independent component analysis (ICA) provided by the *MNE* package was performed to remove ocular artifacts. A copy of the original data was high-pass filtered at 1 Hz cutoff frequency to remove low-frequency drifts. This procedure was followed since studies have shown that ICA works best with data filtered at 1 Hz [64]. The electrode *FP1*—located on the forehead and near the eyes—was used as the EOG channel to regress ocular artifacts out. After identifying the ICA components, we applied the ICA to the previously preprocessed 0.1 Hz and 40 Hz filtered data. The resulting signal was further cleaned using *Autoreject*, allowing for automatic interpolation of bad channels. Finally, we visually inspected the resulting signal to look for bad channels that *Autoreject* did not correct and interpolated them manually using the *interpolatebads* function from *MNE*.

EEG single-trial epochs ranging from 0 ms pre-trigger to 1400 ms post-trigger onset (start trial) and 1000 ms pre-trigger to 400 ms post-trigger onset (end trial) were extracted from the preprocessed data, averaged and evoked potentials calculated for each participant, stimulation condition and experimental phase. Finally, paired contrasts of the experimental phases were calculated by subtracting the baseline activity during the pre-intervention phase from the intervention and post-intervention phases, respectively. These paired contrasts reflect changes in brain activity across time for each stimulation condition/participant and were computed using the *MNE* function ${combined\_evoked}$ with *weights* equal to [1, − 1].

#### Alpha power

We computed the time–frequency representation (TFR) from the evoked potentials to estimate alpha wave power (8–13 Hz) for the time window 0–1400 ms from the start trial and − 1000–400 ms from the end trial across all electrodes for each participant/condition and paired contrast. The Morlet wavelet transform was used to compute the time–frequency representation of the averaged EEG signal, with frequencies ranging from 8 to 13 Hz in 1 Hz steps. The wavelet width of the sliding temporal window was set to 1.5 cycles. The wavelet width of 1.5 cycles was chosen to prioritize temporal precision in our analysis, while still providing adequate frequency resolution [65]. Power was calculated as the sum of the squares of the real and imaginary Morlet components. The square roots of the power values, termed spectral amplitudes (in μV), were then averaged over single trials separately for the start and end trials to yield the total averaged spectral amplitudes for each condition and electrode site [66]. This procedure provides an electric scalp amplitude (μV) map for the signal contained in a specific frequency band informing about the topographic distribution of alpha-wave strength.

#### Global field power

Changes in the strength of the electric field at the scalp were quantified using global field power (GFP; [67–69]). GFP is defined as the standard deviation of the voltages measured at each electrode and time point (as described in [68, 69]) and provides a metric for the global strength of a response independently of the source configuration, i.e., GFP does not depend on the spatial distribution of the electric field. The GFP was computed for the evoked potentials of each participant/condition and paired contrast using the python package *NeuroKit2* [70].

#### Global map dissimilarity

Changes in the configuration of brain networks (i.e., topography) were identified using global map dissimilarity (GMD; [68]). GMD is defined as the root mean square of the difference between two strength-normalized vectors, i.e., the instantaneous voltage potentials across the electrode montage, also known as “maps”. GMD is a metric to quantify topographic differences between two electric fields, regardless of pure amplitude modulations across conditions (i.e., GFP). Since changes in topography necessarily result from differences in the configuration of the brain’s underlying active generators [59, 71], GMD provides a statistical means to determine whether brain networks mediate responses to our texture discrimination task across experimental phases and conditions. The GMD was computed for the evoked potentials of each participant/condition and paired contrast using the python package *NeuroKit2* [70].

### Statistical analysis

#### Behavioral data

The analysis of the behavioral data was conducted in *R* using linear mixed models (LMM), employing the *lmerTest* package [72]. The model (Eq. 7) included the main effect of ***Time*** (*Pre-intervention*, *Intervention*, and *Post-intervention*), ***Group*** (*Sham*, *WH-Stim*), and their interaction. We also accounted for random effects due to individual participant (ID) variability. The *Sham* group and *Pre-intervention* were consistently used as the reference levels for the ***Group*** and ***Time*** factors, respectively.

7 $$\begin{aligned} VI_{i}\sim Group*Time+(1|ID) \end{aligned}$$

We used a LMM for each of the variables of interest ${{\varvec{VI}}}_{i}$, i.e., those related to task performance (probability of correct responses, *PropRes*), and those related to the kinematic exploration metrics, i.e., the scanning time (*ScanTime*), path length (*PathLength*), and scanning speed (*ScanSpeed*).

For each model, we inspected whether they complied with the LMM assumptions using the *R* package *Performance* [73], namely normality of the residuals and homogeneity of variance. We applied a *log* transformation on the dependent variables that were detected as non-normally distributed and rerun the *Performance* analysis to ensure that the data was normally distributed.

Results from the LMM are reported both, uncorrected and corrected according to Benjamini and Hochberg (HB). Statistical significance for all analyses was set to ${\alpha = 0.05}$. When an uncorrected significant interaction between ***Group*** and ***Time*** was detected, we performed the respective planned contrasts analysis: (i) with-in groups differences from *Pre*- to* Post-intervention*, and (ii) with-in groups differences from *Pre-intevention* to *Intervention*. The contrast analysis was performed using the *R* package *Emmeans* [74] with Bonferroni correction.

When displaying the results, we observed that the *WH-Stim* group seemed to perform better than the *Sham* group during *Pre-intervention*. To rule out that our randomization resulted in initial differences between groups in task performance, we performed a *t*-test between groups for the probability of correct responses, *PropRes*.

#### Electrophysiological data

Within-group comparisons across experimental phases for the TFR, GFP, and GMD (i.e., pre-intervention versus intervention and pre-intervention versus post-intervention), were performed using non-parametric cluster-level one-sample *t*-test employed in the *MNE* function ${permutation\_cluster\_1samp\_test}$. More precisely, one-sample *t*-tests were performed using the *Scipy* function ${ttest\_1samp\_no\_p}$ on the paired contrasts to look for significant changes from zero for each group (i.e., *Sham* and *WH-Stim*). A one-sample *t*-test on the paired contrast is equivalent to performing a dependent sample *t*-test between the two phases within each group.

Between-group comparisons across stimulation conditions for the paired contrasts of TFR, GFP, and GMD (i.e., *Sham* versus *WH-Stim* groups) were performed using non-parametric permutation cluster analysis based on independent sample *t*-statistics with the *MNE* function ${permutation\_cluster\_test}$.

The *t*-statics threshold for all permutation tests was set to 2.17, computed using the Scipy’s *stats*.*distributions*.*t*.*ppf* function. The $\alpha$ was set to 0.05 and the degree of freedom (*df*) was equal to the number of participants per group minus one, as we used a two-tailed test. The *t*-threshold calculation can be expressed as:

8 $$\begin{aligned} t_{thresh} = t_{dist}.ppf\left( 1-\frac{\alpha }{2}, df\right) , \end{aligned}$$

where $t_{dist}$ refers to the *t*-distribution, *ppf* is the percent point function, $\alpha$ is the significant level, and *df* represents the degrees of freedom.

For the permutation tests, we used the pre-intervention data as the reference for within-group comparisons, and the Sham group data as the reference for between-group comparisons.

## Results

All participants were able to perform the whole experiment in the allocated session.

### Behavioral results

#### Task performance

**Fig. 5:**
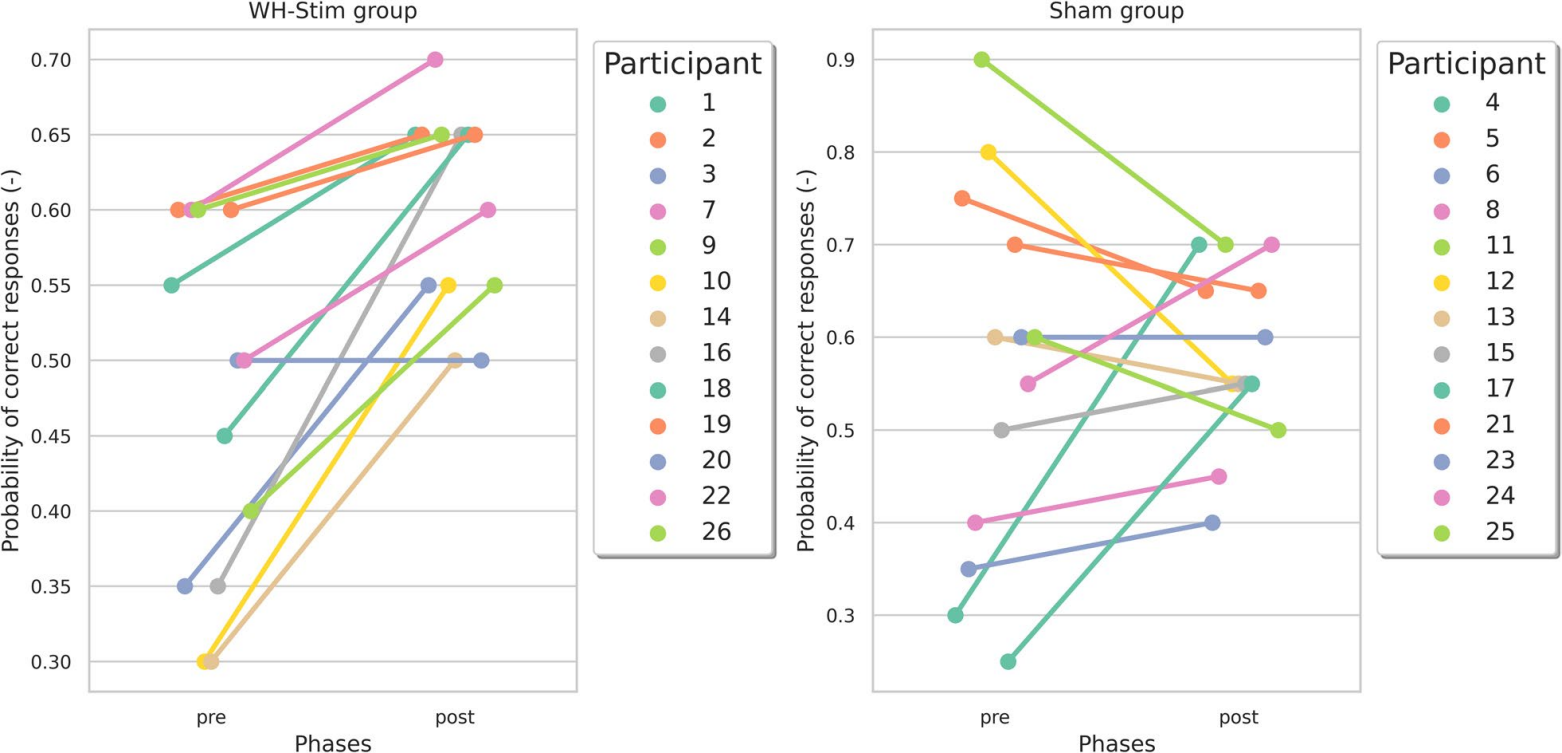
Task performance (i.e., probability of correct responses) at pre- and post-intervention for individual participants in the whole-hand stimulation (left) and sham group (right)

Figure 5 shows the task performance (i.e., probability of correct responses) at pre- and post-intervention for individual participants in the whole-hand stimulation (WH-Stim) and Sham groups. We did not find significant differences in the task performance during the pre-intervention between groups.

Results from the LMM and post-hoc contrasts are summarized in Table 2. We found that participants who trained with the whole-hand stimulation improved their task performance from pre- to post-intervention to a greater extent than the Sham group (Table 2; ${\beta = 0.115}$, ${SE = 0.052}$, ${df = 48.00}$, ${t = 2.201}$, and ${p = 0.033}$). However, this difference did not maintain statistical significance after adjusting for multiple comparisons using the BH correction (${p_{adj} = 0.098}$). The post-hoc analysis revealed a significant improvement in the WH-Stim group from pre- to post-intervention (Table 2; ${p_{adj} = 0.001}$), while the Sham group did not significantly improve after intervention.

**Table 2:** Results from the linear mixed model and planned post-hoc contrast on task performance, i.e., probability of correct responses (*PropRes*)

Variable	Estimate	SE	df	*t* value	*p* (${p_{\text {adj}}}$)
Task performance					
PropRes (–)					
(Intercept)	0.562	0.034	54.920	16.675	<**0.001** (<**0.001**)
Group *WH-Stim*	$-$0.092	0.048	54.920	$-$1.938	0.058 (0.104)
Time *int*	$-$0.053	0.037	48.000	$-$1.418	0.163 (0.195)
Time *post*	0.019	0.037	48.000	0.519	0.606 (0.606)
Group *WH-Stim:Time int*	0.097	0.052	48.000	1.859	0.069 (0.104)
Group *WH-Stim:Time post*	0.115	0.052	48.000	2.201	**0**.**033** (0.098)
Planned contrasts					
Group *Sham*					
*pre*-*int*	0.053	0.037	48.000	1.418	(0.325)
*pre*-*post*	$-$0.019	0.037	48.000	$-$0.519	(1.000)
Group *WH-Stim*					
*pre*-*int*	$-$0.045	0.037	48.000	$-$1.211	(0.463)
*pre*-*post*	$-$0.135	0.037	48.000	$-$3.632	(**0**.**001**)

*SE* standard error, *df* degrees of freedom, *pre* pre-intervention, *int* intervention, *post* post-intervention. The reference level for the **Group** factor is *Sham*. The reference for the **Time** factor is *pre*, the pre-intervention. Significant *p*-values (*p *< 0.05) are indicated in bold

#### Kinematic outcomes

**Fig. 6:**
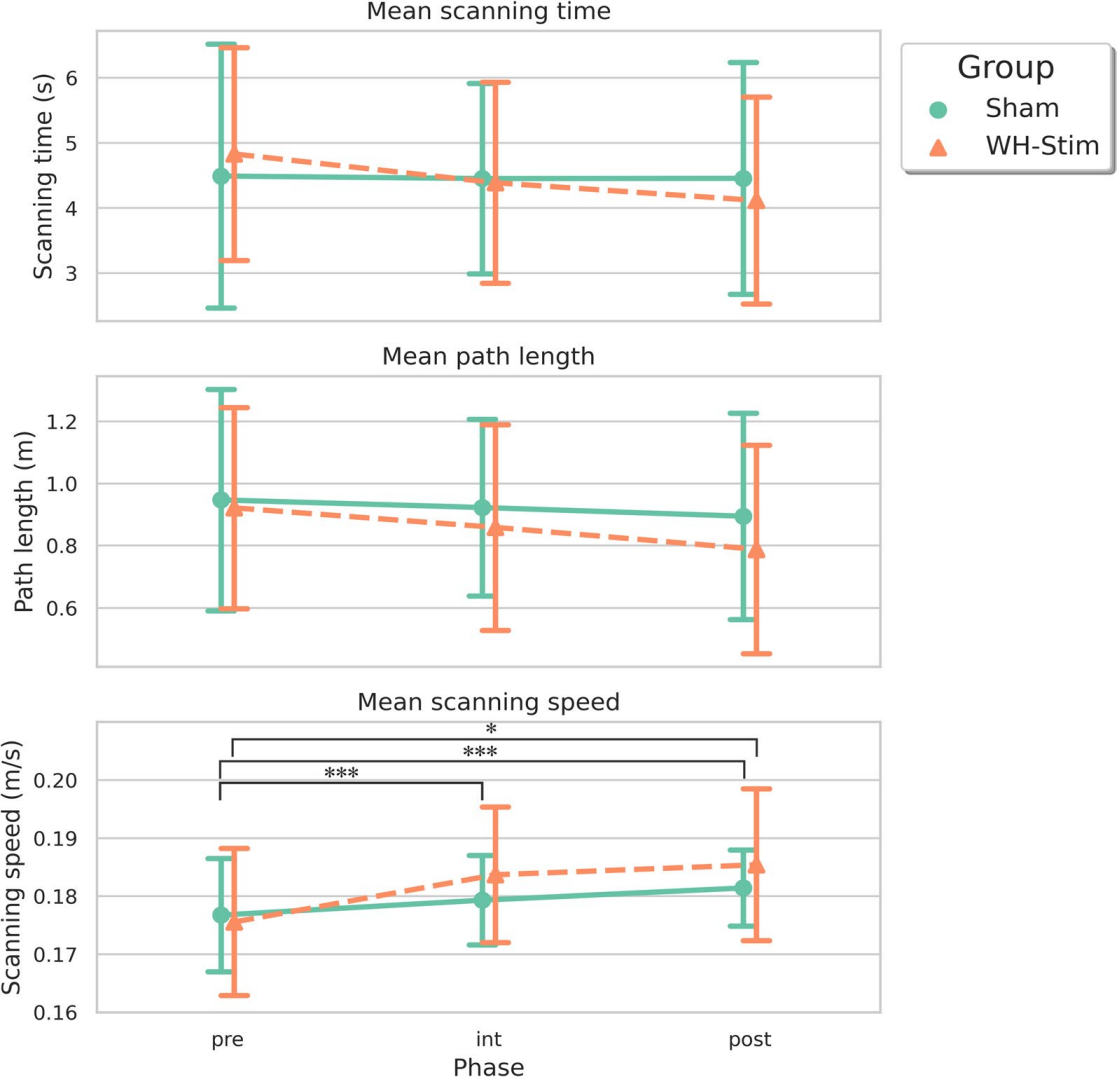
Mean kinematic outcomes—scanning time, path length, and scanning speed—during the different experimental phases for the two experimental groups. The error bars correspond to the standard deviation. ‘$$*$$’ *p* <  0.05, ‘$$***$$’ *p* < 0.001

Figure 6 illustrates the evolution of the kinematic outcomes—namely scanning time, path length, and scanning speed—, during the different experimental phases for the two experimental groups. The results from the LMM and post-hoc contrasts are summarized in Table 3.

We only found differences between groups in the scanning speed. While overall all participants explored the textures significantly faster at post-intervention compared to pre-intervention (Table 3; ${\beta = 0.005}$, ${SE = 0.002}$, ${df = 48.00}$, ${t = 2.727}$, ${p = 0.009}$, ${p_{adj} = 0.027}$), the WH-Stim increased the speed to a larger extend than the Sham group (Table 3; ${\beta = 0.005}$, ${SE = 0.002}$, ${df = 48.00}$, ${t = 2.143}$, ${p = 0.037}$). Yet, this interaction effect did not hold after the BH correction (${p_{adj} = 0.056}$). Similar interaction effects were observed in the scanning speed from pre-intervention to intervention. The post-hoc analysis revealed a significant increase in the scanning speed from pre- to post-intervention in both groups (WH-Stim: $p < 0.001$; Sham $p = 0.018$) and from pre-intervention to intervention in the WH-Stim group ($p < 0.001$) (Fig. 6 and Table 3). No main effects or interaction effects were found in the other kinematic outcomes.

**Table 3:** Results from the linear mixed model and post-hoc contrast on kinematic outcomes

Variable	Estimate	SE	df	*t* value	*p* (${p_{\text {adj}}}$)
Kinematic outcomes					
Log(ScanTime) (s)					
(Intercept)	1.413	0.105	30.998	13.506	<**0.001** (<**0.001**)
Group *WH-Stim*	0.109	0.148	30.998	0.739	0.465 (0.698)
Time *int*	0.026	0.064	48.000	0.414	0.681 (0.817)
Time *post*	0.007	0.064	48.000	0.115	0.909 (0.909)
Group *WH-Stim:Time int*	$-$0.127	0.090	48.000	$-$1.402	0.1673 (0.335)
Group *WH-Stim:Time post*	$-$0.181	0.090	48.000	$-$1.998	0.051 (0.154)
Log(PathLength) (m)					
(Intercept)	$-$0.116	0.106	30.719	$-$1.095	0.282 (0.658)
Group *WH-Stim*	$-$0.030	0.150	30.719	$-$0.199	0.843 (0.869)
Time *int*	$-$0.011	0.064	48.000	$-$0.166	0.869 (0.869)
Time *post*	$-$0.057	0.064	48.000	$-$0.898	0.374 (0.658)
Group *WH-Stim:Time int*	$-$0.070	0.090	48.000	$-$0.781	0.439 (0.658)
Group *WH-Stim:Time post*	$-$0.118	0.090	48.000	$-$1.312	0.196 (0.658)
ScanSpeed (m/s)					
(Intercept)	0.177	0.003	30.345	60.519	<**0.001** (<**0.001**)
Group *WH-Stim*	$-$0.001	0.004	30.350	$-$0.288	0.775 (0.775)
Time *int*	0.003	0.002	48.000	1.500	0.140 (0.168)
Time *post*	0.005	0.002	48.000	2.727	**0.009 (0.027)**
Group *WH-Stim:Time int*	0.006	0.002	48.000	2.300	**0**.**026** (0.052)
Group *WH-Stim:Time post*	0.005	0.002	48.000	2.143	**0**.**037** (0.056)
Planned contrasts					
Group *Sham*					
*pre*-*int*	$-$0.003	0.002	48.000	$-$1.500	(0.280)
*pre*-*post*	$-$0.005	0.002	48.000	$-$2.727	**(0.018)**
Group *WH-Stim*					
*pre*-*int*	$-$0.008	0.002	48.000	$-$4.753	(<**0.001**)
*pre*-*post*	$-$0.010	0.002	48.000	$-$5.758	(<**0.001**)

SE stands for standard error. *SE* standard error, *df* degrees of freedom, *pre* pre-intervention, *int* intervention, *post* post-intervention. The reference level for the **Group** factor is *Sham*. The reference for the **Time** factor is *pre*, the pre-intervention. Significant *p*-values (*p <* 0.05) are indicated in bold

### Electrophysiological results

Inferences and claims for each cluster-based permutation test are reported following the recommendation provided by Sassenhagen and Draschkow [75] and Maris et al. [76].

#### Time–frequency analysis

**Fig. 7:**
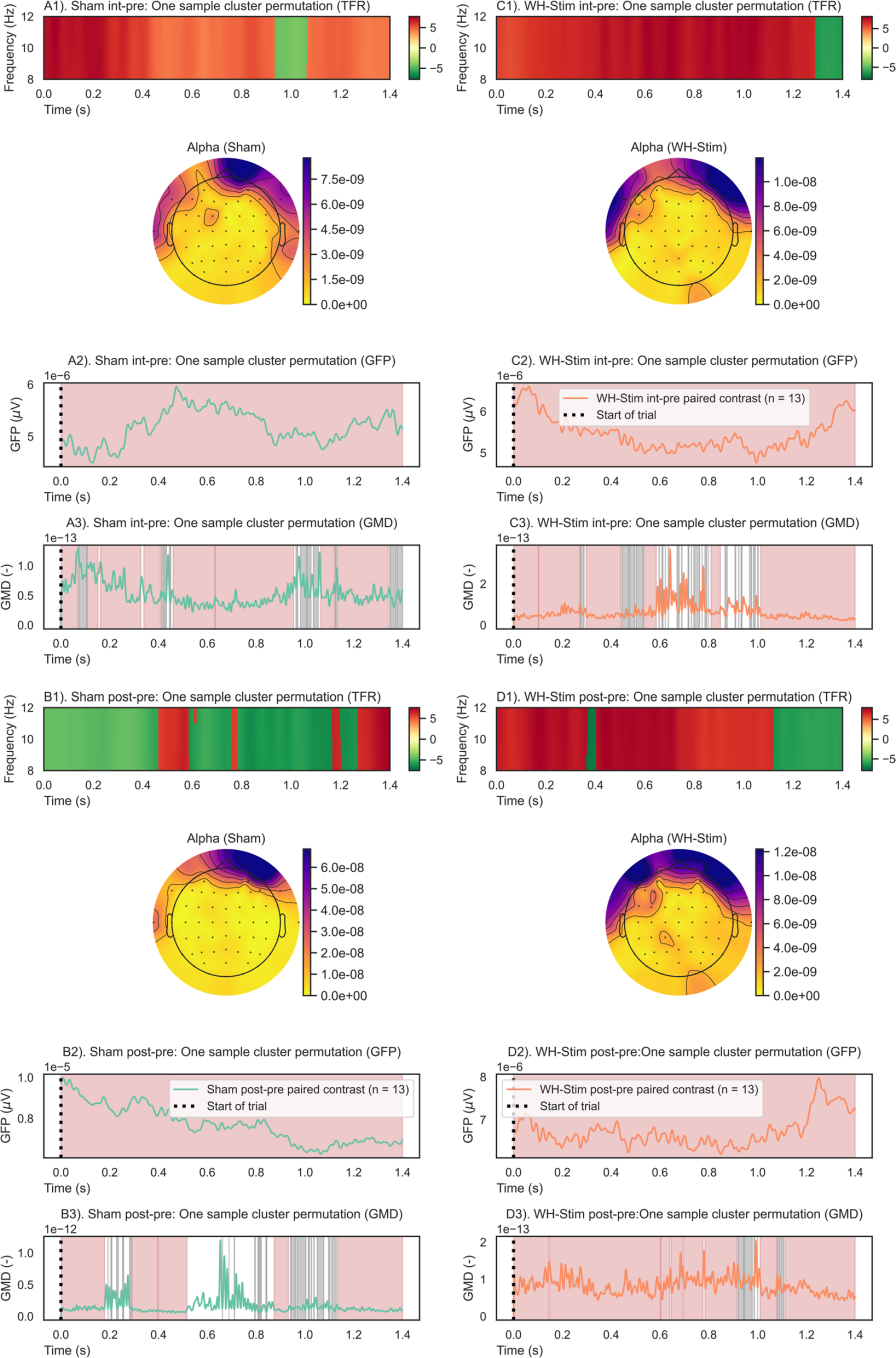
Start-of-trial analysis results of one-sample cluster permutation tests for time–frequency representation (TFR), global field power (GFP), and global map dissimilarity (GMD). Each group and paired contrast was performed separately: A1–3) Sham group paired contrast intervention–pre-intervention; B1–3) Sham group paired contrast post–pre-intervention; C1–3) WH-Stim group paired contrast intervention–pre-intervention; and D1–3) WH-Stim group paired contrast post–pre-intervention. TFR: Positive (red) and negative (green) clusters, $p<$  0.05. GFP and GMD: Significant clusters (red) $p<$ 0.05, non-significant clusters (grey) $p>$ 0.05

**Fig. 8:**
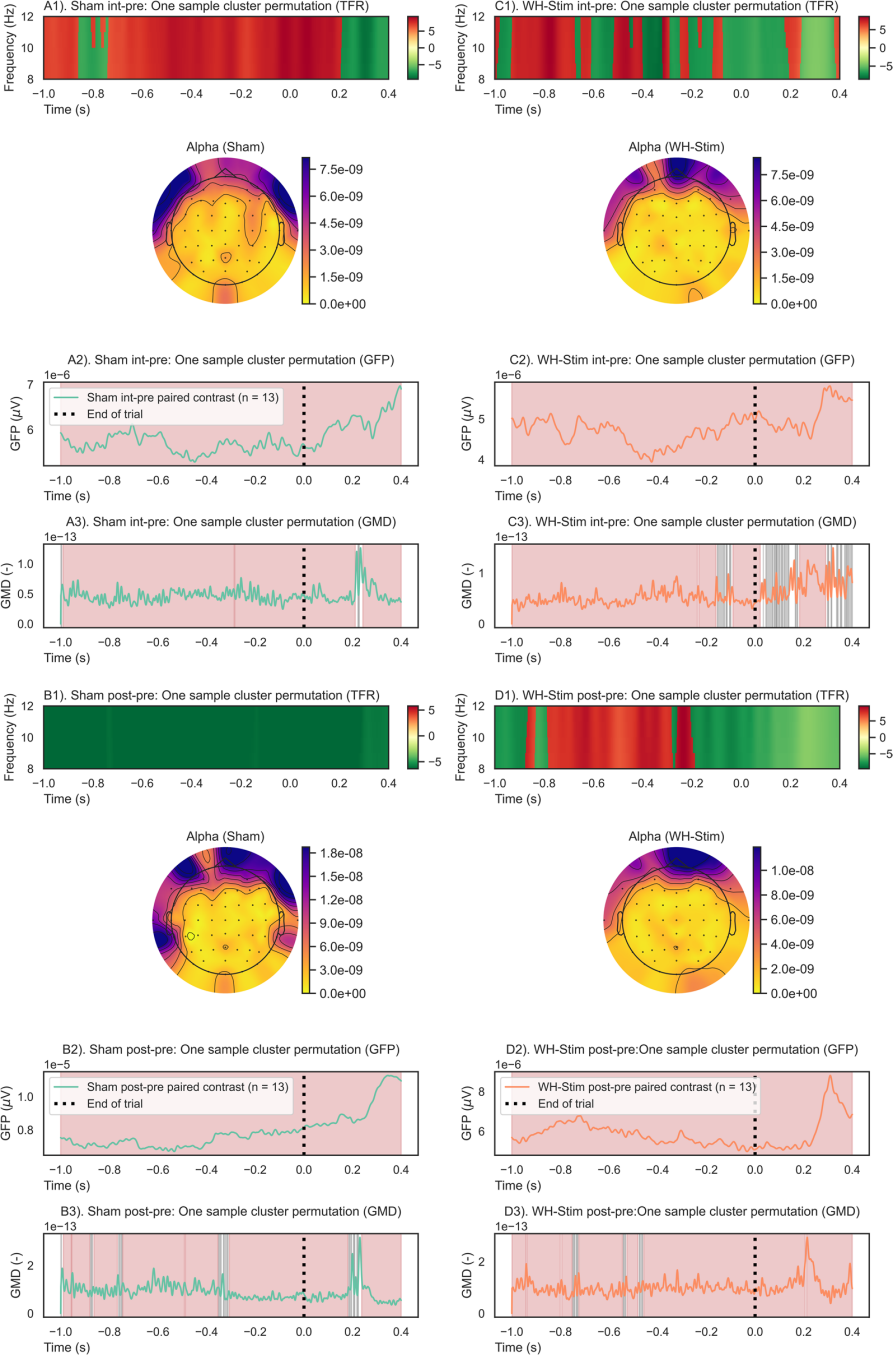
End-of-trial analysis results of one-sample cluster permutation tests for time–frequency representation (TFR), global field power (GFP), and global map dissimilarity (GMD). Each group and paired contrast was performed separately: A1–3) Sham group paired contrast intervention–pre-intervention; B1–3) Sham group paired contrast post–pre-intervention; C1–3) WH-Stim group paired contrast intervention–pre-intervention; and D1–3) WH-Stim group paired contrast post–pre-intervention. TFR: Positive (red) and negative (green) clusters, $p<$ 0.05. GFP and GMD: Significant clusters (red) $p<$ 0.05, non-significant clusters (grey) $p>$ 0.05

**Fig. 9:**
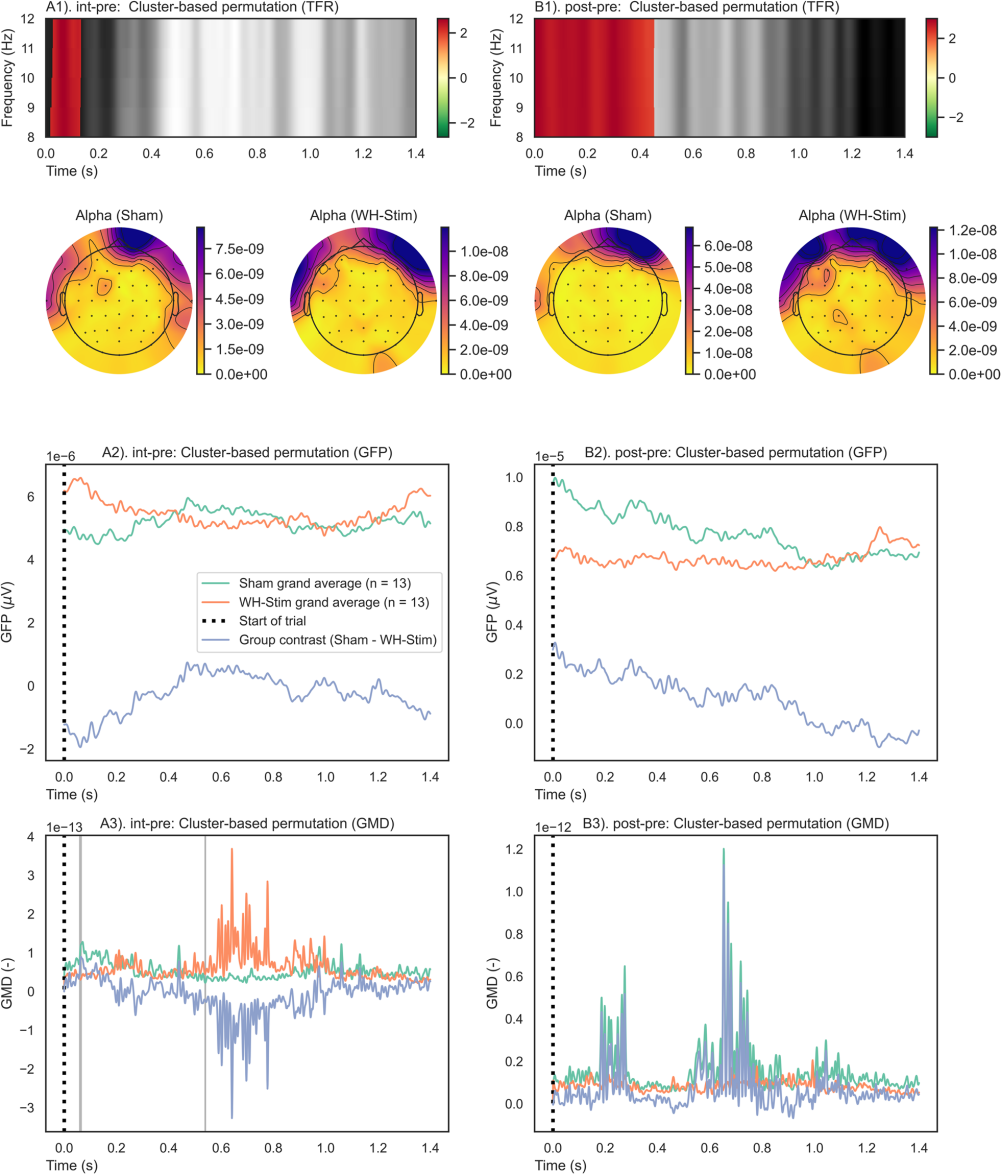
Between group comparisons of time–frequency representations (TFRs), global field powers (GFPs), and global map dissimilarities (GMDs) for the start trial. A permutation cluster test was performed between the groups’ TFRs, GFPs, and GMDs. The time window was from 0.0 s (i.e., the start of the trial) to 1.4 s after it. Each paired contrast was performed separately between the groups: A1–3) Paired contrasts intervention–pre-intervention between Sham vs. WH-Stim; B1–3) Paired contrasts post–pre-intervention between Sham vs. WH-Stim. TFR: Positive (red) and negative (green) clusters, p< 0.05. GFP and GMD: Significant clusters (red) $p<$ 0.05, non-significant clusters (grey) $p>$ 0.05

**Fig. 10:**
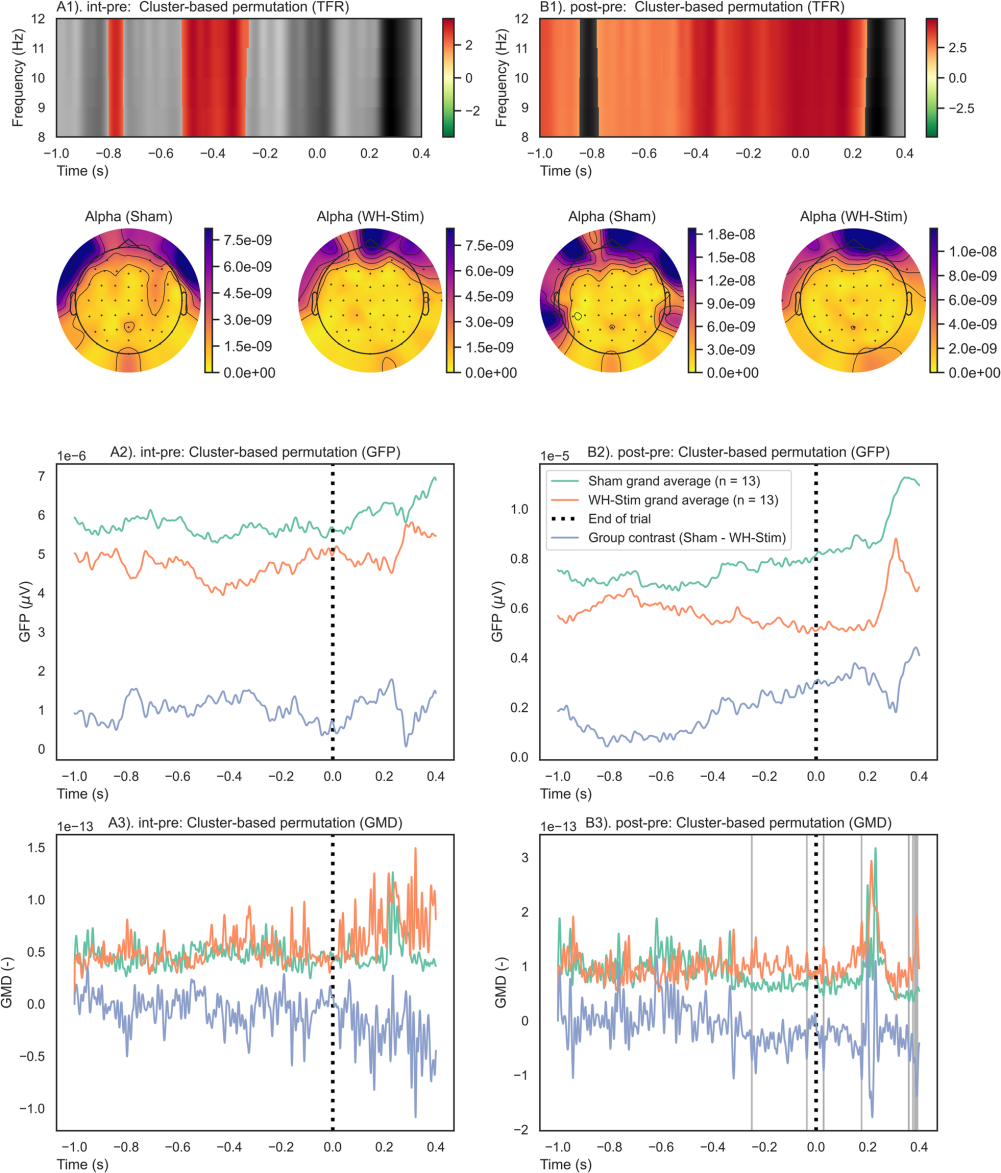
Between group comparisons of time–frequency representations (TFRs), global field powers (GFPs), and global map dissimilarities (GMDs) for the end trial. A permutation cluster test was performed between the groups’ TFRs, GFPs, and GMDs. The time window was from − 1.0 s (i.e., 1 s before the onset of the end trial) to 0.4 s after it. Each paired contrast was performed separately between the groups: A1–3) paired contrasts intervention–pre-intervention between Sham vs. WH-Stim; B1–3) paired contrasts post–pre-intervention between Sham vs. WH-Stim. TFR: Positive (red) and negative (green) clusters, $p<$ 0.05. GFP and GMD: Significant clusters (red) $p<$ 0.05, non-significant clusters (grey) $p>$ 0.05

An overview of the results is presented in Figs. 7, 8, 9, and 10. A positive cluster (red shade) indicates an increase in alpha power in the contrast of interest (e.g., from pre- to post-intervention) relative to the reference, i.e., pre-intervention and Sham group. In contrast, a negative cluster (green shade) indicates a decrease in alpha power in the contrast of interest relative to the reference, i.e., pre-intervention and Sham group.

#### Start of the trial

The one-sample cluster-based permutation tests on the paired phase contrasts resulted in a significant difference in alpha power change within each group’s *int-pre* and *post-pre*, as shown in Fig. 7.

Alpha activity significantly increased in the Sham group during the intervention (changes *int-pre*), with more prominent changes occurring from 0.0 to 0.4 s after starting the trial (Sub-Fig. 7A). On the other hand, during post-intervention, the Sham group exhibited a significant decrease in alpha power relative to the pre-intervention, with more prominent changes occurring after 0.8 s of starting the trial (Sub-Fig. 7B). For the WH-Stim group, our results show a significant increase in alpha power after both intervention and post-intervention phase relative to pre-intervention, with most prominent changes lasting around half a second and appearing after 0.2 s after trial start (Sub-Fig. 7C, D).

Permutation cluster tests on the paired contrasts between groups revealed significant differences in alpha activity changes at the intervention relative to pre-intervention (Sub-Fig. 9A) and post-intervention relative to pre-intervention (Sub-Fig. 9B). In particular, the WH-Stim group showed a stronger increase in alpha activity when starting the texture discrimination trial during intervention and post-intervention than the Sham group. The significant clusters at intervention and post-intervention relative to pre-intervention lasted 0.1 and 0.4  s, respectively. However, due to the nature of the cluster-based permutation test, we cannot be certain about the exact time when the significant cluster occurs, only about the duration of the alpha power differences over time.

#### End of the trial

Figure 8 illustrates the time–frequency evaluation outcomes for the end of the trial analysis. Results of the one-sample cluster-based permutation tests conducted separately for each group on their paired phase contrasts showed that alpha activity significantly increased in the Sham group during the intervention (changes *int-pre*), with most prominent changes occurring from − 0.6 to 0.2 s after texture discrimination (Sub-Fig. 8A). On the other hand, during post-intervention texture discrimination, the Sham group exhibited a pronounced and significant decrease in alpha power relative to the pre-intervention (Sub-Fig. 8B). For the WH-Stim group, we found a significant alpha power increase after both intervention and post-intervention phase relative to pre-intervention early during texture discriminating (− 0.8 to − 0.2 s), while later in the time window, the alpha activity decreased relative to the pre-intervention (Sub-Fig. 8C, D).

Permutation cluster tests on the paired phase contrasts at the end of the trial revealed significant differences in alpha activity changes between the groups after the intervention relative to pre-intervention (Sub-Fig. 10A) and post-intervention relative to pre-intervention (Sub-Fig. 10B). The WH-Stim group showed a stronger increase in alpha activity when discriminating textures during the intervention and especially during the post-intervention relative to pre-intervention than the Sham group. The significant clusters after the intervention and post-intervention lasted 0.2 and 1.0  s, respectively.

#### Global field power (GFP)

For the start of the trial, one-sample permutation tests showed that both groups had significant differences in the paired contrasts compared to zero (*p* < 0.05) (as shown in Fig. 7), indicating a significant increase in GFP values from pre-intervention to the intervention and from pre-intervention to post-intervention. However, permutation-based cluster tests revealed no significant differences between the group paired contrasts, as can be seen in Fig. 9, where no significant clusters were observed between groups for either *int-pre* or *post-pre*.

Similarly, for the end of the trial, one-sample permutation tests showed significant changes in GFP paired contrasts compared to zero (*p* < 0.05), indicating significantly higher GFP during intervention and post-intervention relative to pre-intervention for both groups (shown in Fig. 8), with no significant differences between the group paired contrasts (Fig. 10).

#### Global map dissimilarity (GMD)

At the start of the trial, overall GMD values were significantly modulated for both groups, indicative of an engagement of different brain networks during the intervention and post-intervention relative to the pre-intervention. Further, comparison between groups did not show significant modulation of engaged brain networks, as shown in Fig. 9.

Similarly, at the end of the trial, engaged brain networks were significantly modulated during the intervention and post-intervention relative to pre-intervention, as shown in Fig. 7. Comparison between groups did not show significant modulation of engaged brain networks apart from very short clusters which did not reach significance during the *post-pre* paired contrast, as shown in Fig. 9.

## Discussion

In this study, we aimed to investigate the effects of sensory electrical stimulation (SES)—using whole-hand stimulation delivered at 95% of participants’ individual sensory thresholds—, on sensory training using a well-controlled robotic experimental set-up, compared to sham stimulation. In a single-session study with 26 healthy participants, we evaluated the participants’ performance during a passive sensory training task that involved the tactile discrimination of three virtual visually identical textures and their texture exploration behavior during the task. Additionally, to study the neural underpinnings of SES, we assessed brain signals associated with sensory processing during the tactile discrimination task, namely alpha power, the strength of the brain signal, and changes in engaged brain networks.

### Effects of sensory electrical stimulation combined with sensory training on task performance and exploration behavior

We hypothesized that both groups, the one who received whole-hand stimulation and the Sham group, would exhibit improvements in texture discrimination during and post-intervention relative to pre-intervention. We found that the integration of whole-hand sensory stimulation with sensory training indeed improves task performance—i.e., the probability of correct responses—after the intervention. However, we did not observe a significant improvement in the discrimination of textures between pre-intervention and intervention. Contrary to our expectations, the Sham group did not show significant improvements in the probability of correct responses neither between pre-intervention to intervention nor pre- to post-intervention. This is contrary to the enhanced performance after training observed in a previous study we performed with thirty-six healthy participants who trained in a similar system without sensory electrostimulation [50]. However, in our previous study, the training/intervention included 120 trials, twice the number included in this study. Thus, the number of trials in this study might have not been sufficient for the participants in the Sham group to significantly enhance their performance after training.

We also hypothesized that participants who received whole-hand stimulation during training would demonstrate significantly better texture discrimination during and after the intervention, relative to pre-intervention, compared to those who did not receive stimulation. However, we did not find significant differences between groups in the improvements in task performance from pre- to post-intervention.

The lack of significant difference in task performance within phases in the Sham group and between groups could be attributed to the large variability observed in the Sham group performance at pre- and post-intervention, as observed in Fig. 5. While some participants in the Sham group indeed improved their performance in the texture discrimination task after the intervention, especially those with a lower probability of correct responses during pre-intervention, participants who initially performed better at pre-intervention seemed to decline their performance after the intervention. While ceiling effects could be expected in initially better performers, as there is little room for improvement in those initially more skilled participants, this does not explain their performance degradation after the intervention. Potential rationales might be that they became especially fatigued after training [77] or felt unmotivated to further improve their performance. Although we did not find significant differences in task performance during pre-intervention between groups, the random allocation of participants into the two groups resulted in four participants with an initial probability of correct responses above 0.6 in the Sham group, while no participants with a performance above this value were allocated into the whole-hand stimulation group. Increasing the sample size in future studies might help to better evaluate the effect of initial skill level on the effectiveness of sensory training and sensory electrostimulation on texture discrimination.

Regarding the texture exploration behavior, we found that participants in the stimulation group increased significantly the scanning speed from pre-intervention to intervention and to post-intervention. Together with the significant increase in task performance found in the stimulation group, these exploration behavior changes might reflect higher confidence in the task in hand in participants in the stimulation group. The changes in scanning speed between phases in the stimulation group were significantly different from those in the Sham group, whose participants only showed a significant increase in the scanning speed after intervention. These results complement previous findings in tactile discrimination research, which have shown that texture perception remains constant regardless of exploration speed, yet, sensory stimulation has been reported to enhance movement kinematics, e.g., movement speed, in stroke patients [78]. The differences in exploration behavior between groups support the hypothesis that whole-hand stimulation seems to enhance texture discrimination performance after sensory training compared to training without stimulation.

### Effects of sensory electrical stimulation combined with sensory training on brain networks

Our study demonstrated that sensory training induces changes in underlying brain networks. Both groups showed enhanced electrical activity (i.e., global field power; GFP and significant changes in topographies (i.e., global map dissimilarity; GMD)—indicative of engagement of different brain networks—during and after the intervention relative to the pre-intervention. We suggest these results point towards a modulation of attentional resources and (short-term) neural plasticity or reorganization, elicited by sensory training.

Our particular focus was alpha power as a well-established neural correlate of sensory processing [48]. Alpha power has been associated with sensory processing efficiency and attention [79]. In Li et al.’s work, alpha power is considered a good indicator of neural excitability [80]. Alpha power fluctuations have been found to correlate with task performance during complex sensory information processing [80]. Moreover, Brickwedde et al. emphasized that high levels of somatosensory alpha oscillations are essential for efficient performance during perceptual tasks [81]. As hypothesized, the sensory training significantly impacted the engagement of alpha oscillations, especially in those participants in the whole-hand stimulation group, and in line with previous literature [48, 81]. While alpha power generally increased in both groups during the intervention, alpha power decreased during post-intervention in the Sham group and increased in the stimulation group relative to pre-intervention. Especially during the end of the trials, participants who received stimulation showed a pronounced increase in alpha activity from pre- to post-intervention compared to participants who did not receive stimulation, suggesting that attention was enhanced during the whole decision-making process [79, 82]. Our results on enhanced alpha power during and after the sensory training suggest that the sensory discrimination task improved sensory processing and attention, especially when combined with electrical stimulation.

Global field power can be considered a reference-free metric quantifying the “hilliness” (i.e., the variability) of the potential landscape derived from EEG data. Low GFP reflects a flat potential distribution, i.e., desynchronization of neural activity, whereas high GFP indicates an increase in synchronous neural activity [68, 69]. Changes in GFP have previously been associated with modulations in attention and processing resources allocated to sensory stimuli [83, 84]. Our GFD results suggest that sensory training enhanced participants’ attention during and after training relative to pre-intervention, and sensory training led to changes in brain networks in both groups, while no differences between groups were found. Our results align with previous research showing that somatosensory training induces brain plasticity. For example, Sarasso et al. found an association between sensory discrimination training and the lateralization of brain activity in sensorimotor areas during sensory and motor tasks in healthy participants [85]. Thus, while the observed sensory training effects in task performance in the Sham group were less pronounced than initially hypothesized, EEG correlates indicate consistent changes in the allocation of attentional resources and short-term reorganization in sensory brain networks associated with the sensory discrimination training in both groups.

The neurophysiological effects related to sensory training and electrical stimulation were similar for the start and end trials, i.e., the preparation and decision-making process respectively. However, a smaller increase in alpha power across experimental phases was noted at the end of the trial when visually compared to the start of the trial. This observed difference, which was not evaluated with statistical analysis, might relate to the end trial signifying the decision process (i.e., the 1 s before pressing the decision button) and task completion (i.e., 0.4 s after pressing the button), and thus physical relaxation and potentially reduced cognitive demands and, consequently, a smaller alpha power increase [48]. In contrast, during the start of the trials, participants likely remained engaged in the task for the entire duration of the analyzed EEG sequence/epoch. Yet, we cannot draw definitive conclusions, as the two types of epochs were not statistically compared.

Notably, we observed that alpha power was sensitive to the effects of sensory electrical stimulation. The stimulation group exhibited a significantly larger increase in alpha power from pre-intervention to intervention and post-intervention than the Sham group, likely due to the effects of sensory electrical stimulation on the central nervous system. Whole-hand sensory electrostimulation stimulates cutaneous and muscle afferents, improving sensation, cortical activity [22], and neural excitability [86]. Moreover, sensory electrical stimulation is thought to induce plasticity in the central nervous system through use-dependent, long-term potentiation-like mechanisms [87–89]. Although the precise mechanisms of how sensory electrical stimulation enhances sensory processing remain unclear, it has been proposed that the observed increase in alpha power changes in the whole-hand stimulation group may indicate a link between electrical stimulation and improved sensory processing [90]. In our study, enhancing sensory processing through sensory electrical stimulation might have resulted in improved task performance (touch sensibility) as observed in the whole-hand stimulation group. Alternatively, the increase in alpha power might be associated with attentional effects, which can also enhance alpha power. However, the behavioral results in our study show a consistent trend for better performance in the stimulation group, implying a meaningful influence of the intervention. Furthermore, no differences in GFP were found between groups, a metric sensitive to the overall effort/attention during a task [91]. Finally, the global map dissimilarity results suggest no differences in the engagement of underlying brain networks between groups. This finding suggests that electrical stimulation enhances sensory processing in statistically indistinguishable sensory networks compared with no stimulation. Further research is needed to elucidate the precise mechanisms through which sensory electrical stimulation impacts alpha power and somatosensory processing and their implications for neurorehabilitation while accounting for potential confounding factors such as attentional effects.

In conclusion, our results suggest that robotic-aided sensory training with and without electrical stimulation enhances attention toward sensory stimuli and induces short-term changes in the organization of underlying networks. Adding electrical stimulation during sensory training increases the engagement of more specialized brain networks associated with sensory processing (i.e., alpha oscillations), potentially enhancing performance. Further studies with larger sample sizes are recommended to validate these findings.

### Study limitations

We acknowledge several study limitations that merit discussion. The primary limitation of our study, which might contribute to the lack of significant differences between groups, is the small sample size. Although the sample size was derived from a power analysis based on our previous experiment, this experiment involved slightly different comparison stimuli. We might have underestimated the participants’ variance with the new comparison stimulus set.

A second limitation is related to the starting and ending points of the trials. We did not control whether participants were within the haptic table at the start and end of the trials. Participants could have started the trial on a smooth surface (outside a texture), or on a texture. Similarly, participants might have selected the response once they were outside a texture or moving inside the texture. This is relevant because roughness might be a confounding factor that could modulate alpha power activity [92].

## Conclusion

We investigated neurophysiological responses to somatosensory electrical stimulation during a robotic-aided texture discrimination task to shed light on the potential of whole-hand electrical stimulation together with robotic-assisted sensory training in promoting sensory retraining. We found that robotic-aided sensory training together with whole-hand electrical stimulation improves the discrimination of virtual textures associated with short-term adaptations in underlying brain networks. While there was an improvement in task performance after the intervention in the sensory electrical stimulation group, the differences between groups did not reach significance. Yet, we observed that the whole-hand stimulation group significantly moved faster while exploring the textures after training and showed a significantly stronger increase in the engagement of specialized sensory-related brain areas after training compared to the group that trained without electrical stimulation. Further research is needed, particularly with brain-injured patients, to confirm the potential benefits of combined sensory electrical stimulation and sensory retraining in neurorehabilitation.

## Data Availability

The data used to draw the conclusions in this paper can be found in the [Zenodo] repository, https://doi.org/10.5281/zenodo.8032879.
